# Neighborhood environmental deprivation predicts the generalized neurocognitive deficit in schizophrenia

**DOI:** 10.1016/j.schres.2025.12.006

**Published:** 2025-12-19

**Authors:** Luyu Zhang, Zhixin Zhang, Gregory P. Strauss

**Affiliations:** Department of Psychology, University of Georgia, United States

**Keywords:** Schizophrenia, Cognition, Environmental deprivation, Rurality

## Abstract

**Background:**

The generalized neurocognitive deficit is a core feature of schizophrenia (SZ). However, no current treatments have demonstrated satisfactory efficacy in remediating cognitive impairment in this population. This lack of treatment progress might be due in part to the failure to consider broader environmental factors and social determinants of health. The current paper explored the contributing role of these factors to the generalized neurocognitive deficit in SZ.

**Methods:**

Participants included 114 outpatients with SZ and 117 matched healthy controls (CN) who completed a cross-sectional study. Cognitive impairment was assessed by the MATRICS Consensus Cognitive Battery (MCCB). Environmental deprivation was measured by Area Deprivation Index (ADI). Rurality was measured by the rural urban continuum code (RUCC). A linear regression model was fitted to predict MCCB total score with ADI, RUCC, and the interaction between ADI and RUCC as predictors.

**Results:**

The overall model was significant, *F* = 8.06, *p* < .001, *R^2^* = 0.14. The effect of ADI score was also significant, *β* = −0.36, *t* = −2.30, *p* < .05; while the effect of RUCC, *β* = 0.06, *t* = 0.34, *p* = .73, and the interaction between ADI and RUCC, *β* = −0.001, *t* = 0.27, *p* = .99, were nonsignificant.

**Conclusions:**

Neighborhood-level environmental deprivation predicted cognitive impairment in SZ. This highlights the need to incorporate environment-focused interventions when treating cognitive impairment in SZ.

## 1. Introduction

Individuals with SZ reliably demonstrate impairment across most cognitive domains at a magnitude of ~1.5SD below healthy controls, which has been referred to as the generalized neurocognitive deficit (GND) in SZ (Dickinson et al., 2004, 2006; Dickinson and Harvey, 2009; Heinrichs and Zakzanis, 1998). This feature has been consistently associated with poorer clinical outcomes, including lower recovery rates (Kopelowicz et al., 2005), worse quality of life (Savilla et al., 2008), greater odds of future hospitalization (Kadakia et al., 2022), higher levels of unemployment (Marwaha and Johnson, 2004), and worse social functioning (Cohen et al., 2006). Although cognitive impairments are a critical clinical target in SZ therapeutics, limited efficacy has been demonstrated with current treatments (Green et al., 2019; Horan et al., 2022). First and second-generation antipsychotics were found to improve cognitive functioning, but the effect was small and of questionable clinical significance (Heinrichs, 2007; Keefe et al., 2007). Traditional psychosocial treatment for social cognition appeared to be effective, but existing programs are limited by factors such as poor generalization to functional improvements (Horan and Green, 2019). More recent cognitive remediation programs have demonstrated some promising results van Duin et al., 2019; Kambeitz-Ilankovic et al., 2019; Prikke et al., 2019; Vita et al., 2021, 2023). These programs usually have four core features: facilitation by a therapist, cognitive exercises, procedures to develop problem-solving strategies, and procedures to facilitate transferring skills to real world functioning (Bowie et al., 2020). While responsibilities differ by program, the role of the therapist usually includes collaborative goal setting, cognitive problem formulation, and targeted domain identification. Cognitive exercises are usually completed via computer-based training programs that allow participants to engage repetitively with stimuli that are associated with targeted cognitive domains. Procedures to develop problem-solving strategies focus on developing “meta-cognitive skills” which focuses on developing the ability to monitor cognitive skill implementation while working toward goals on each cognitive task. Lastly, procedures to facilitate transfer to real world functioning are designed to help patients generate specific activities that they can link to the training activities with the goal of applying their cognitive skills in daily life. This is usually completed collaboratively with a therapist. Unfortunately, access to these interventions is limited and treatment benefits are often short-lived after training is completed (Calzavara-Pinton et al., 2024; Tripathi et al., 2018). Similarly, although management of cardiovascular diseases (e.g., diabetes, hypertension) and other lifestyle factors (e.g., sleep difficulties) is commonly recommended (McCutcheon et al., 2023), these strategies are intended to minimize the cognitive decline associated with these additional risk factors, instead of targeting the SZ disease process itself.

One possible explanation for this lack of treatment progress for cognitive impairments in SZ is the failure to consider how broader environmental processes and social determinants of health (SDoH) contribute to cognitive impairment in SZ. There are 5 main tenants of SDoH - economic stability, education access and quality, healthcare access and quality, neighborhood and built environment, and social and community context (Marmot et al., 2008; U.S. Department of Health and Human Services, 2020). Many neighborhood-level SDoH factors have been found to increase the likelihood of experiencing cognitive decline in older adulthood (Walsh et al., 2025). These factors include homelessness and housing instability (Babulal et al., 2022; Roncarati et al., 2024), neighborhood deprivation (Becerril et al., 2023; Choi et al., 2024; Dai et al., 2023; Li et al., 2023a, 2023b; Lai et al., 2023; Ou et al., 2024; Zhao et al., 2021), exposure to noise (Cantuaria et al., 2021; Hu et al., 2023; Huang et al., 2021; Zhao et al., 2021), neighborhood disorder (Wong and Wang, 2022), exposure to toxins (Jalilian et al., 2018; Killin et al., 2016; Yan et al., 2016; Zhao et al., 2021), and air pollution (Livingston et al., 2024). Similar associations between neighborhood environmental deprivation and worse cognition have also been found in neurological disorders, such as epilepsy (Busch et al., 2023; Reyes et al., 2024) and Alzheimer~s disease and related dementias (Besser et al., 2024; Majoka and Schimming, 2021; Rahemi et al., 2025). However, this relationship remains largely understudied in SZ.

Research has also found that there is a higher prevalence rate of neurological diagnoses (e.g., mild cognitive impairment, dementia) in rural areas compared to urban areas (Li et al., 2023a; Liu et al., 2024). Several factors have been identified to potentially account for this difference, including less education (Kim et al., 2019; Marcopulos et al., 1997), lower socioeconomic status (Bartley et al., 2023; Kim et al., 2019), and more vascular risk factors/diseases (Nunes et al., 2010; Peritogiannis et al., 2022). Interestingly, several studies highlighted neighborhood-level factors that could explain the higher prevalence rates in rural areas. For example, Weden et al. (2018) argued that fewer opportunities for social engagement in the neighborhood and less developed medical, public health, and social service infrastructure in rural areas might contribute to the difference in prevalence rates. Similarly, Ali (2024) found that people living in rural communities generally have less access to specialty healthcare services, which would result in inadequate management of symptoms. However, these studies did not explicitly investigate whether rurality interacted with neighborhood-level environmental resources in contributing to cognitive impairment. While people living in rural areas tend to have fewer environmental resources, the distinction between neighborhood deprivation and rurality is meaningful as they are conceptualized differently (Butler and Beale, 1994; Singh, 2003) and have been shown to differentially impact health-related outcomes (Balio et al., 2024). Additionally, previous studies investigating cognition in rural populations primarily focused on healthy controls and people with dementia. Therefore, it remains unclear how living in rural areas, both independently and interactively with neighborhood-level environmental deprivation, contributes to cognitive impairment in SZ.

The current study aims to explore the contribution of both neighborhood-level environmental deprivation and rurality to the generalized neurocognitive deficit in SZ. Neighborhood-level environmental deprivation was measured by Area Deprivation Index (ADI), and rurality was measured by the rural-urban continuum code (RUCC). The ADI provides rank scores for the overall environmental deprivation in relation to the whole state (i.e., ADI state score) and the whole country (i.e., ADI national score) using census block data (Kind & Buckingham., 2018). It incorporates factors that are commonly thought to impact health, including education, employment, housing quality, and poverty measures. Higher ADI scores (i.e., more environmental deprivation) have been linked to cognitive impairment in older adults at risk for dementia (Gogniat et al., 2023, 2025), as well as negative symptoms and underlying reward processing deficits in SZ (Strauss et al., 2025). RUCC classifies U.S. counties into 9 categories that distinguish metropolitan and nonmetropolitan areas by population size, level of urbanization, and adjacency to urban areas (Butler and Beale, 1994; U.S. Department of Agriculture, 2013). It is widely used to study rural–urban disparities in health, socioeconomic, and environmental outcomes (Edwards et al., 2023; Hall et al., 2006; McCall-Hosenfeld et al., 2014; Townley et al., 2017).

The following 3 hypotheses were made. First, more neighborhood-level environmental deprivation as indexed by a higher ADI score would predict more severe GND in SZ. Second, living in rural areas as indexed by a higher RUCC would predict more severe GND in SZ. Third, the interaction between environmental deprivation as indexed by the ADI and rurality as indexed by the RUCC would predict more severe GND in SZ.

## 2. Methods

### 2.1. Participants

Participants included 114 outpatients meeting DSM-5 criteria for a psychotic disorder (SZ) and 117 matched CN. Individuals with SZ were recruited from local community mental health centers and online or printed advertisements. Diagnosis was established via the Structured Clinical Interview for DSM-5 (SCID-5; First, 2015). All participants denied lifetime history of neurological disorders (e.g., epilepsy, traumatic brain injuries) or substance use disorders (other than tobacco). CN did not meet criteria for any current psychiatric disorder. CN also denied any family history of psychosis among first-degree relatives and did not meet lifetime criteria for a psychotic disorder.

SZ and CN groups did not significantly differ on age, parental education, sex, or race; however, CN had significantly higher personal education as expected (see Table 1).

### 2.2. Procedures

Data collection occurred between 2016 and 2019, and all data was collected cross-sectionally. All participants provided informed consent before any study procedures. SCID-5 (First, 2015) was administered to establish diagnosis. MATRICS Consensus Cognitive Battery (MCCB; Nuechterlein et al., 2008) was administered to assess cognitive impairment. Demographic data and home addresses were also collected.

### 2.3. Measures

The MCCB is a commonly used battery for assessing cognitive impairment in SZ. It consists of 10 subtests and provides age- and sex-corrected T-scores for both global cognition and 7 individual cognitive domains, including: processing speed, attention/vigilance, working memory, verbal learning, visual learning, reasoning and problem solving, and social cognition. The MCCB total score served as the index for generalized neurocognitive deficit and was the main dependent variable for all analyses.

Neighborhood-level environmental deprivation was measured by the ADI, which incorporates factors that are commonly thought to impact health, including: education, employment, housing quality, and poverty measures. ADI scores were extracted from the interactive mapping site (https://www.neighborhoodatlas.medicine.wisc.edu/) developed by the University of Wisconsin-Madison (Kind and Buckingham, 2018). Addresses were coded at the census block level. Census block groups (i. e., neighborhoods) created based on participant home addresses were linked to state and national ADI rank scores. For the current study, state ADI score at the time of cognitive assessment was used as the main variable of interest, as national ADI score has been found to be more likely impacted by regional inequalities and therefore misrepresenting neighborhood-level deprivation (Azar et al., 2023; Morenz et al., 2024). Results from analyses using national ADI score can be found in supplemental materials.

Rurality was measured by the RUCC, which classifies U.S. counties into 9 categories that distinguish metropolitan and nonmetropolitan areas by population size, level of urbanization, and adjacency to urban areas. RUCC was extracted from the U.S. Department of Agriculture datasets (https://www.ers.usda.gov/data-products/rural-urban-continuum-codes). Home addresses were coded at the county level using ZIP codes, which were then linked to a specific RUCC category ranging from 1 (most urban) to 9 (most rural).

### 2.4. Data analysis

After inspecting skewness and kurtosis, it was determined that distributions were sufficiently normal for parametric statistical analyses to be performed. All analyses were conducted in R Studio (Posit Team, 2025).

Preliminary analyses included one-way ANOVAs assessing group differences (SZ vs CN) on MCCB total score, MCCB domain scores, ADI state score, and RUCC.

To evaluate the main hypotheses, a linear regression model was conducted to predict MCCB total score. Predictors included ADI state score, RUCC, and the interaction between ADI state score and RUCC.

Several exploratory analyses were also conducted. First, personal education was entered as an additional predictor in the linear regression model (ADI x RUCC x personal education) to account for its effect on cognition. Second, group was entered as an additional predictor in the linear regression model (ADI x RUCC x group). Third, bivariate correlations between ADI state score and MCCB domain scores, as well as between RUCC and MCCB domain scores were conducted. Fourth, all preliminary, main, and exploratory analyses were repeated with ADI national score.

## 3. Results

**Fig. 1.**
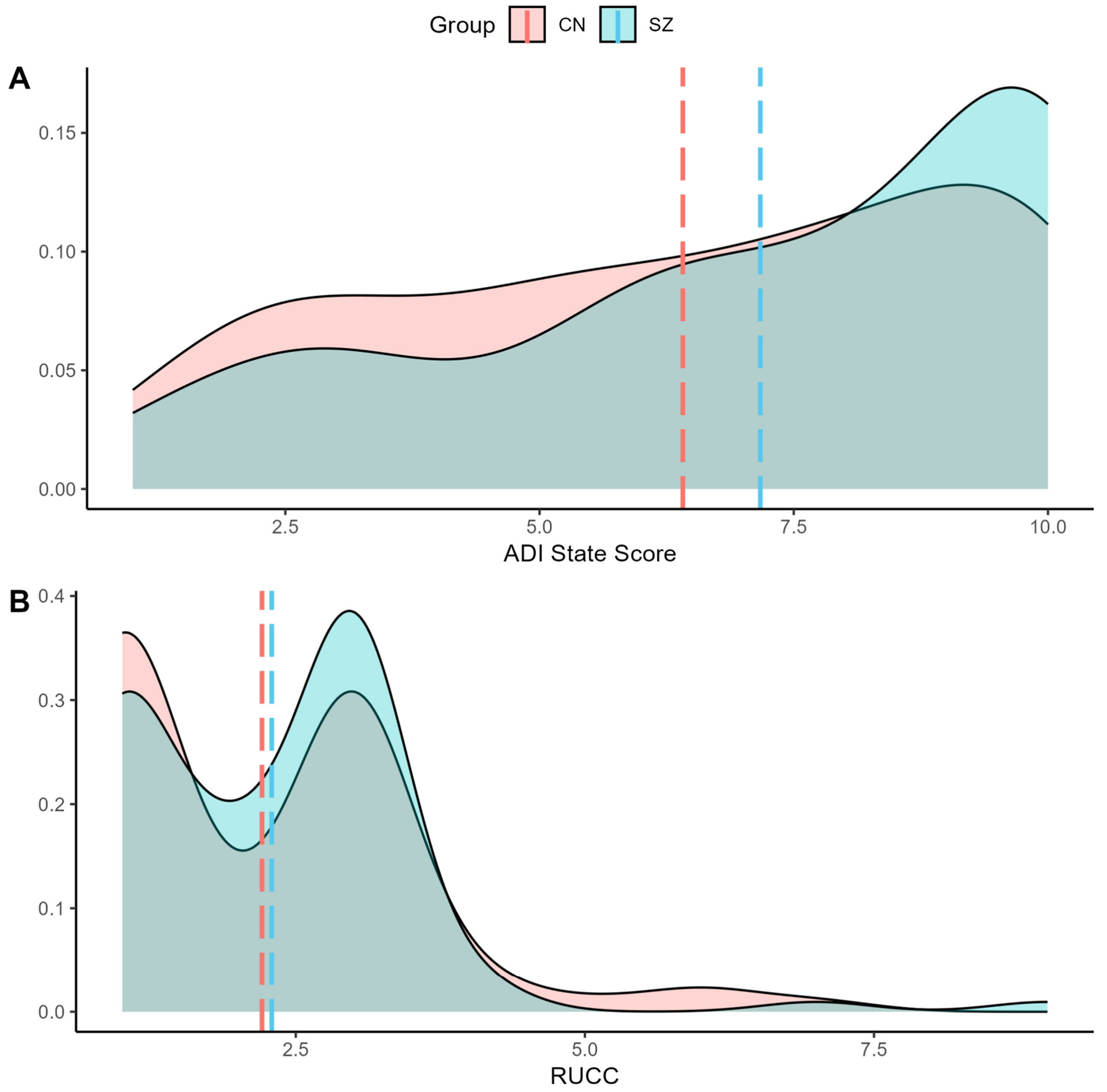
Density Plots of ADI State Score and RUCC in SZ and CN Groups. Dotted Lines represent group means. ADI = Area Deprivation Index, RUCC = Rural Urban Continuum Codes, SZ = Schizophrenia, CN = Healthy Control. Higher ADI score reflects more environmental deprivation. High RUCC suggests residing in more rural areas.

Consistent with previous literature, preliminary analyses showed that compared to CN, SZ had significantly lower MCCB total and domain scores (see Table 2). SZ also had significantly higher ADI state scores, *F* = 7.90, *p* < .05, *d* = 0.27, suggesting that SZ had more environmental deprivation than CN. The group difference on RUCC was nonsignificant, *F* = 0.17, *p* = .68, *d* = 0.07, suggesting that SZ and CN resided in similar areas regarding rurality. Fig. 1 presents the distribution of state ADI score and RUCC by group.

Results from the linear regression model indicated that the overall model was significant, *F* = 8.06, *p* < .001, *R^2^* = 0.14. The effect of ADI state score was significant, *β* = −0.36, *t* = −2.30, *p* < .05; while the effect of RUCC, *β* = 0.06, *t* = 0.34, *p* = .73, and the interaction, *β* = −0.001, *t* = 0.27, *p* = .99, were nonsignificant.

**Fig. 2.**
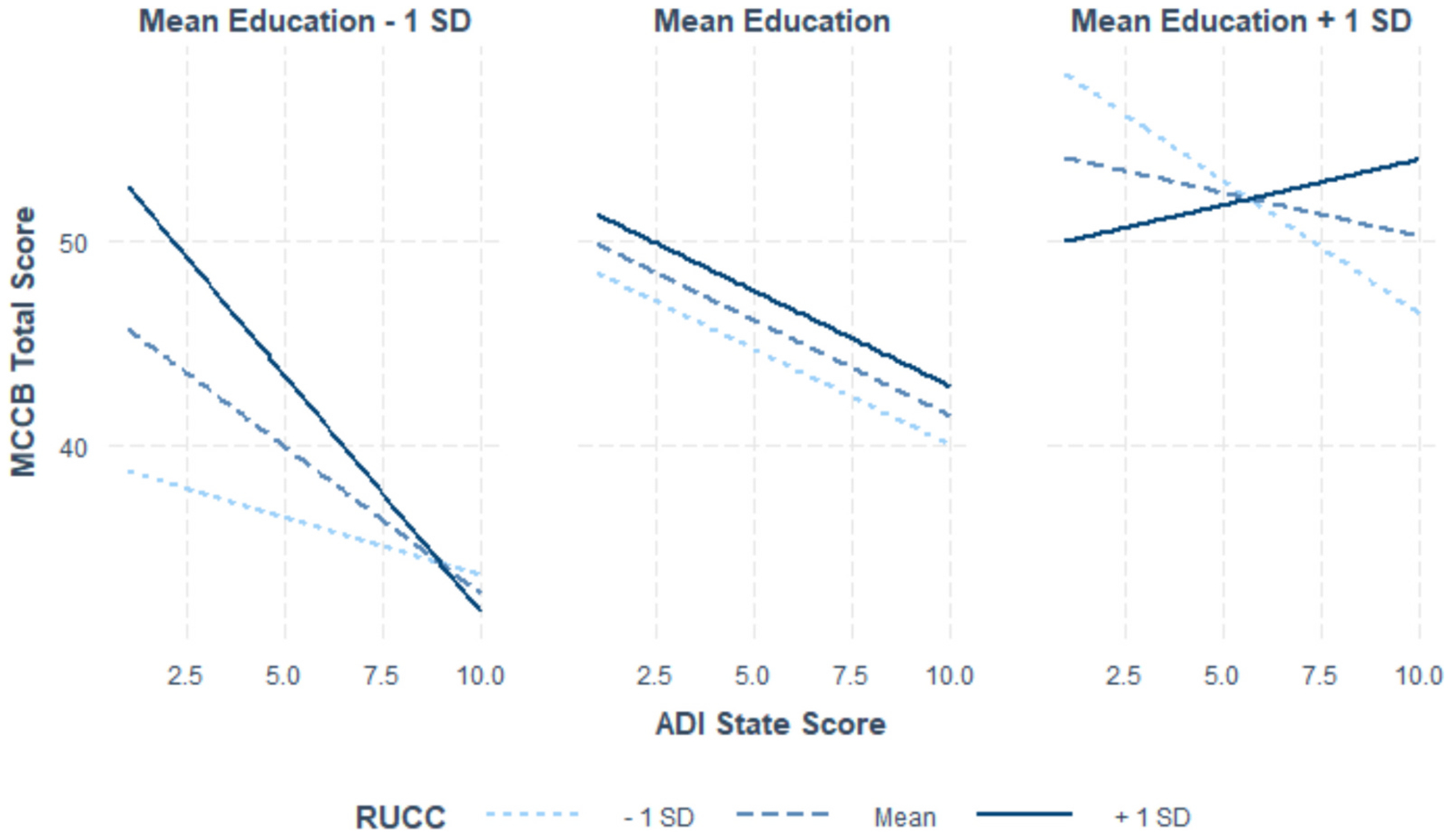
The Three-way Interaction among ADI, RUCC, and Personal Education in Predicting the Generalized Neurocognitive Deficit. ADI = Area Deprivation Index. MCCB = MATRICS Consensus Cognitive Battery. RUCC = Rural Urban Area Code. Higher ADI score reflects more environmental deprivation. High RUCC suggests more rurality.

Detailed results of exploratory analyses can be found in supplemental materials. Briefly, the overall regression model when personal education and the three-way interaction were entered as additional predictors was significant, *F* = 14.4, *p* < .001, *R^2^* = 0.42. Additionally, the three-way interaction among ADI, RUCC, and personal education was also significant, *β* = 0.06, *t* = 2.31, *p* < .05. As shown in Fig. 2, the association between RUCC and MCCB total score varied depending on both ADI state score and personal education. When personal education was at the average level (i.e., 14.1 years), higher ADI score (i.e., more deprivation) predicted lower cognition regardless of the level of rurality. When personal education was one standard deviation (i.e., 2.51 years) below the mean, the effect of ADI score was larger when rurality was higher. When personal education was one standard deviation above the mean, higher ADI scores only predicted lower cognition when rurality was average or lower. Collectively, these results suggested that when personal education was low (i.e., less than 12 years, or the equivalent of a high school diploma), living in rural areas might exacerbate the negative impact of environmental deprivation on overall cognition.

Group as an additional predictor did not meaningfully change the results of the main regression model. SZ group also had significantly lower significant ADI national scores than CN. Regression models using ADI national score as a predictor did not meaningfully differ from those where ADI state score was used. Lastly, the correlations between ADI scores and MCCB scores were all statistically significant, except for correlations involving the Reasoning and Problem-solving domain score (the magnitude of r values was strongest for the Working Memory domain score and weakest for Reasoning and Problem-solving); while only the correlation between the Reasoning and Problem-solving domain score and RUCC was significant. See Table 3 for the full correlation matrix.

## 4. Discussion

As hypothesized, neighborhood-level environmental deprivation significantly predicted the generalized neurocognitive deficit in SZ. This finding aligns with existing literature in healthy controls (Becerril et al., 2023; Choi et al., 2024; Dai et al., 2023; Li et al., 2023a, 2023b; Lai et al., 2023; Ou et al., 2024; Zhao et al., 2021) and neurological disorders such as epilepsy (Busch et al., 2023; Reyes et al., 2024) and Alzheimer’s disease (Besser et al., 2024; Majoka and Schimming, 2021; Rahemi et al., 2025). Contrary to hypotheses, rurality and the interaction between environmental deprivation and rurality did not predict the generalized neurocognitive deficit in SZ in the main analyses. However, results from the exploratory analyses suggested that personal education moderates the relationship among environmental deprivation, rurality, and the generalized neurocognitive deficit. Specifically, if someone does not complete high school, living in rural areas would exacerbate the negative effect environmental deprivation has on overall cognitive functioning. This is generally consistent with the literature on healthy aging populations or older adults with dementia (Kim et al., 2019; Miller et al., 2023; Peritogiannis et al., 2022; Rahman et al., 2021). Results from these studies suggested that in rural environments, additional risk factors may contribute to further cognitive impairment, including less education, higher prevalence of cardiovascular diseases, and less medical and social services (Ali, 2024; Kim et al., 2019; Miller et al., 2023; Nunes et al., 2010; Peritogiannis et al., 2022; Rahman et al., 2021; Weden et al., 2018). The main reason for the discrepancy between the results from the main analyses and the previous literature is likely that most SZ participants from the current study, although not from traditional metropolitan areas (e.g., New York City, Atlanta), still resided in relatively urban areas as defined by the RUCC. The literature on RUCC has generally considered RUCC ≤3 as urban areas and RUCC >3 as rural areas (Delavar et al., 2019; Miller et al., 2023; Wang, Albaroudi and Chen, 2021; Yu, 2019). Previous studies focusing on older adults with healthy aging or dementia generally had samples consisting of more balanced RUCC distribution from most urban (RUCC = 1) to most rural (RUCC = 9) (Miller et al., 2023; Rahman et al., 2021). By comparison, the majority of participants from the current study (approximately 93 %) lived in areas with RUCC ≤3. This highlights the need to recruit more SZ living in rural areas (RUCC >3) for future studies. It is likely that those SZ participants are facing similar additional risk factors as older adults residing in similar areas and therefore are at higher risk for additional cognitive impairment.

Collectively, the findings from the current study extend prior research indicating that broader environmental and SDoH factors contribute to cognitive impairment in SZ. Specifically, previous studies have generally focused on the contributions of other SDoH categories such as economic stability, education access, and social community and context (Aas et al., 2011; Ferraro et al., 2025; Green et al., 2015; Wells et al., 2020). The current study adds to this literature that neighborhood and built environment factors – another main SDoH category, also predict the generalized neurocognitive deficit in SZ. Several potential pathways might explain how neighborhood deprivation contributes to cognitive impairment in SZ, many of which involve the interactions with other SDoH factors. Individuals with SZ may be born into impoverished environments due to their parents and/or close family members having the same disorder. Growing up in such resource deprived areas might limit their access to education and intellectually stimulating activities, both of which are protective against cognitive decline in later life (Livingston et al., 2024). Consequently, these predisposed disadvantages might interfere with normal brain and cognitive development in childhood and adolescence (Barch, 2022; Cardenas-Iniguez et al., 2022).

As cognitive impairment continues to develop into adulthood, individuals with SZ might be constrained to certain types of jobs that lack complexity. This might lead to further cognitive decline due to the negative impact on cognitive reserve, as seen in other clinical populations such as adults with HIV and older adults with mild cognitive impairment (Then et al., 2014; Vance et al., 2015). Additionally, limited job options might restrict their financial resources. As a result, patients with schizophrenia might remain in resource deprived areas as adults, which continues to expose them to specific factors that have shown to contribute to cognitive impairment such as noise and toxins (Cantuaria et al., 2021; Jalilian et al., 2018). Additionally, neighborhood deprivation might also exacerbate other psychiatric symptoms such as negative symptoms (Zhang et al., 2024), which would likely interact with cognitive deficits, further restricting SZ to living in impoverished areas. Lastly, living in impoverished environments throughout life might also limit their access to healthcare services. This will likely delay the management of their cognitive deficits, while also increasing the likelihood of developing other medical conditions that could cause further cognitive decline in later life such as diabetes (Ali, 2024).

These neighborhood-level environmental contributions to cognitive impairment in SZ may warrant distinct interventions. In the aging literature, many have argued that higher-level structural and policy changes focusing on environmental contribution to cognitive decline might be necessary in the prevention and intervention of dementia. For example, in the most recent report of *Lancet* Commission on dementia, Livingston et al. (2024) highlighted the importance of environment-focused strategies for dementia intervention, such as ensuring timely access to medical and social services, and reserving enough community space for physical and social activities. In a comprehensive review on population-level approaches to the prevention of dementia, Walsh et al. (2023) emphasized several population-level strategies that would promote cognitive health, including investing in walking/cycling infrastructure that makes exercise safer and easier, investing in more green spaces, providing adequate training for job positions with complexity, improving internet access (especially in rural areas) to ensure access to telehealth services, and implementing policy changes to regulate the amount of toxins that are released in local areas. Given that findings from current study suggested the contributing role of neighborhood deprivation, similar environment-focused intervention approaches are also likely beneficial for the treatment of cognitive impairment in SZ.

Several limitations need to be considered. First, while the ADI provides a composite estimate of the overall environmental deprivation at the census block level, it has been argued that the index might be primarily driven by a few subcomponents (Hannan et al., 2023). Therefore, it does not provide detailed information about which specific environmental factors contribute to cognitive impairment in SZ. Future studies could dissect the individual elements of the ADI and explore the specific contributions of its sub-indices. For example, using 9 different ADI sub-indices, Ku et al. (2024) found that only neighborhood-level single-parent household was associated with abnormal right hippocampal volume among children. It might be helpful to adopt a similar approach to investigate associations between ADI sub-indices and different cognitive domain scores in SZ in the future. Second, all SZ participants in this study were individuals in the chronic phase. Importantly, given that cognitive impairments are present across all phases of illness in this population (McCutcheon et al., 2023), it will be necessary to examine whether the observed associations generalize to other stages of this disorder. Third, the directionality of the association between cognition and environmental deprivation remains unexplored due to the cross-sectional design. It will be important to investigate this association longitudinally to identify whether environmental deprivation during early development predicts poorer cognitive functioning later in life in SZ. Furthermore, additional research is needed to examine alternate social cognition domains, as several other elements may be crucial for building relationships and navigating the environment (e.g., theory of mind, facial affect labeling). Lastly, the current analyses could not address the length of time a participant lived at their home location and whether this duration impacts cognition.

Despite these limitations, the current study highlighted the role of neighborhood-level environmental deprivation to cognitive impairment in SZ. Given this association, environment-focused interventions might be necessary in treating cognitive impairment in SZ. While rurality did not predict cognitive impairment in the current study, future studies should re-examine this relationship using samples with a larger proportion of SZ living in more rural areas. Additionally, a multitude of biological factors associated with SDOH that are driven by global exposome-level environmental factors (e.g., social epigenetics, allostatic load, accelerated inflammaging, brain structural and functional changes, neurochemical abnormalities, neuroplasticity) are known to affect central nervous system functioning (Jeste et al., 2023). Importantly, the effects of these environmental processes on the brain have a high degree of overlap with global neural mechanisms underlying the GND in SZ (e.g., decreased grey matter volume, white matter abnormalities, imbalance between excitatory and inhibitory neurotransmitters, metabolism, oxidative stress, inflammation) (Dickinson and Harvey, 2009; McCutcheon et al., 2023). Future studies are needed that assess both cognition and neurobiology concurrently with environmental processes to directly test hypotheses about the biological processes mediating the association between cognition and environmental processes using a prospective longitudinal design.

## Supplementary Material

1

## Figures and Tables

**Table 1:** Demographic Characteristics.

	SZ (*n* = 114)	CN (*n* = 117)	Test Statistics
Age	39.19 (12.49)	38.94 (11.75)	*F* = 0.02, *p* = .876
Sex (%female)	53.6	56.3	*X^2^* = 0.06, *p* = .798
Personal Education	12.98 (2.03)	15.20 (2.46)	*F* = 53.98, *p* < .001
Parental Education	13.59 (2.79)	13.90 (2.56)	*F* = 0.75, *p* = .389
Race/Ethnicity (%)			*X*^2^ = 4.59, *p* = .333
White	69.1	62.5	
Black	20.00	18.80	
LatinX	4.50	8.90	
Asian	0.90	4.50	
Other	5.40	5.40	

Note. SZ = Schizophrenia, CN = Healthy Control.

**Table 2:** Group Differences on MATRICS Consensus Cognitive Battery (MCCB) Scores.

	SZ (n = 114)	CN (n = 117)	Test Statistics	Cohen’s *d*
Total Score	37.77 (12.41)	49.68 (10.33)	*F* = 61.58***	1.0
Processing Speed	42.00 (11.05)	50.87 (10.13)	*F* = 40.48***	0.84
Attention/Vigilance	41.03 (11.49)	48.96 (9.85)	*F* = 30.91***	0.75
Working Memory	42.46 (13.47)	50.97 (11.11)	*F* = 27.50***	0.69
Verbal Learning	41.01 (10.77)	46.93 (9.44)	*F* = 19.78***	0.59
Visual Learning	39.60 (13.53)	48.44 (11.20)	*F* = 29.21***	0.71
Reasoning and Problem Solving	45.89 (11.32)	50.03 (10.07)	*F* = 8.64**	0.39
Social Cognition	44.71 (12.00)	51.31 (10.61)	*F* = 19.48***	0.59

Note: All scores are age- and gender- corrected T-scores. Standard deviations are provided in the parentheses.

** *p* < .01

*** *p* < .001.

**Table 3:** Spearman Correlations between ADI Scores and MCCB Domain Scores.

	ADI State Score	ADI National Score	RUCC
Processing Speed	−0.28***	−0.25***	0.06
Attention/Vigilance	−0.26***	−0.24***	0.08
Working Memory	−0.36***	−0.33***	0.04
Verbal Learning	−0.33***	−0.34***	0.09
Visual Learning	−0.26***	−0.26***	0.12
Reasoning and Problem Solving	−0.08	−0.13	−0.01
Social Cognition	−0.29***	−0.27***	0.18*
Total Score	−0.39***	−0.38***	0.10

Note: ADI = Area Deprivation Index. MCCB = MATRICS Consensus Cognitive Battery. RUCC = Rural Urban Area Code.

* *p* < .05,

*** *p* < .001.

Higher ADI score reflects more environmental deprivation. High RUCC suggests residing in more rural areas.

## Appendix A. Supplementary data

Supplementary data to this article can be found online at https://doi.org/10.1016/j.schres.2025.12.006.
