# Supplementary material for: Neighborhood environmental deprivation predicts the generalized neurocognitive deficit in schizophrenia
Source: Schizophr Res. Author manuscript; Available in PMC 2026 Jun 30. (PMC13318238; doi:10.1016/j.schres.2025.12.006)
Supplement: 1 [file NIHMS2187926-supplement-1.docx]

**Supplemental Materials**

NEIGHBORHOOD ENVIRONMENTAL DEPRIVATION PREDICTS THE GENERALIZED NEUROCOGNITIVE DEFICIT IN SCHIZOPHRENIA

Luyu Zhang, M.A., M.S.

Zhixin Zhang, B.S.

Gregory P. Strauss, Ph.D. *

Department of Psychology, University of Georgia

*Corresponding Author: Gregory P. Strauss, Ph.D., Email: gstrauss@uga.edu.

Phone: +1-706-542-0307. Fax: +1-706-542-3275. University of Georgia, Department of Psychology, 125 Baldwin St., Athens, GA 30602.

Results of the exploratory analyses that are not reported in the main manuscript are detailed below.

**MCCB Total Score Predicted by ADI x RUCC x Group**

The overall regression model when group (CN as the reference group) and the three-way interaction were entered as additional predictors was significant, *F* = 10.6, *p* < .001, *R^2^* = .33. The effects of the following predictors were significant: ADI state score, *β* = -.46, *t* = -2.32, *p* < .05 and group, *t* = -2.73, *p* < .01.

**Analyses with ADI National Score as the Main Variable of Interest**

Results from one-way ANOVA indicated that SZ had significantly higher ADI national score compared to CN, *F* = 6.39, *p* < .05, *d* = .34, suggesting that SZ had significantly more environmental deprivation.

All linear regression models in the main and exploratory analyses were also fitted using ADI national score as the predictor. When ADI national score,RUCC, and their interaction were entered into the model, the overall model was significant, *F* = 8.29, *p* < .001, *R^2^* = .14. ADI national score was the only significant predictor, ADI national score, *β* = -.40, *t* = -2.48, *p* < .05. When ADI national score, RUCC, personal education, and the three-way interaction were entered as the predictors, the overall model was significant, *F* = 14.28, *p* < .001, *R^2^* = .41. There were several significant predictors, including RUCC, *β* = 2.94, *t* = 2.24, *p* < .05; personal education, *β* = 1.05, *t* = 2.37, *p* < .05; ADI national score x RUCC, *β* = -.004, *t* = -2.17, *p* < .05; RUCC x personal education, *β* = -.38, *t* = -2.22, *p* < .05; and the three way interaction, *β* = .05, *t* = 2.16, *p* < .05. When ADI national score, RUCC, group, and the three-way interaction were entered as the predictors, the overall model was significant, *F* = 10.04, *p* < .001, *R^2^* = .32. The effects of the following predictors were significant: ADI national score, *β* = -.42, *t* = -1.99, *p* < .05, and group, *t* = -2.04, *p* < .05.
